# Aerobic and Anaerobic Biodegradation of 1,2-Dibromoethane by a Microbial Consortium under Simulated Groundwater Conditions

**DOI:** 10.3390/ijerph16193775

**Published:** 2019-10-08

**Authors:** Qing Wang, Miaoyan Yang, Xin Song, Shiyue Tang, Lei Yu

**Affiliations:** 1 Key Laboratory of Soil Environment and Pollution Remediation, Institute of Soil Science, Chinese Academy of Sciences, Nanjing 21008, China; qwang@issas.ac.cn (Q.W.); yangmiaoyan@g.ecc.u-tokyo.ac.jp (M.Y.); sytang@issas.ac.cn (S.T.); 2 University of Chinese Academy of Sciences, Beijing 100049, China; 3 Department of Environmental Engineering, Nanjing Forestry University, Nanjing 210037, China; lyu@njfu.edu.cn

**Keywords:** 1,2-dibromoethane, biodegradation, microbial consortium, co-substrates, rhamnolipid, aerobic and anaerobic conditions

## Abstract

This study was conducted to explore the potential for 1,2-Dibromoethane (EDB) biodegradation by an acclimated microbial consortium under simulated dynamic groundwater conditions. The enriched EDB-degrading consortium consisted of anaerobic bacteria *Desulfovibrio*, facultative anaerobe *Chromobacterium*, and other potential EDB degraders. The results showed that the biodegradation efficiency of EDB was more than 61% at 15 °C, and the EDB biodegradation can be best described by the apparent pseudo-first-order kinetics. EDB biodegradation occurred at a relatively broad range of initial dissolved oxygen (DO) from 1.2 to 5.1 mg/L, indicating that the microbial consortium had a strong ability to adapt. The addition of 40 mg/L of rhamnolipid and 0.3 mM of sodium lactate increased the biodegradation. A two-phase biodegradation scheme was proposed for the EDB biodegradation in this study: an aerobic biodegradation to carbon dioxide and an anaerobic biodegradation via a two-electron transfer pathway of dihaloelimination. To our knowledge, this is the first study that reported EDB biodegradation by an acclimated consortium under both aerobic and anaerobic conditions, a dynamic DO condition often encountered during enhanced biodegradation of EDB in the field.

## 1. Introduction

One of the most commonly detected contaminants in groundwater is 1,2-Dibromoethane (EDB), because of its extensive addition to leaded gasoline and soil fumigants several decades ago [1]. EDB is highly toxic and probably carcinogenic in humans, and it can lead to damage to the lungs, liver, kidneys, stomach, reproductive system, respiratory system, and nervous system in mammals [2]. EDB can be characterized by the relative hydrophilia, poor bioavailability, and extremely low natural attenuation rate (t_1/2_ = 17.33 years) in groundwater [3]. Today, EDB can still persist in groundwater at high levels at some contaminated sites, even though its usage was banned in gasoline and agriculture in the 1980s in Canada, Sweden, and the USA [1], and later followed by countries such as Japan, Israel, and Australia [4], as well as China, Belize, Colombia, Cyprus, Ecuador, Kenya, and Argentina [5,6]. For example, a survey conducted in South Carolina revealed that 10% of underground-storage-tank sites had EDB concentrations greater than 200 μg/L. Additionally, the median concentration of EDB at these sites was 5 μg/L, which was two orders of magnitude higher than the maximum contaminant level (MCL) of 0.05 μg/L [3,7]. 

In view of its common existence and persistence in groundwater and its detrimental effects on human health, various methods are employed to remove and decompose EDB in groundwater. Previous studies showed that EDB can be degraded successfully by several chemical methods, such as zero valent iron reduction [8], sulfate reduction [9,10], and photocatalytic oxidation [11]. However, these methods have their limitations, such as high cost and the negative impacts of the chemicals on the ecosystem. Therefore, there is a need to develop cost-effective techniques to address the EDB contamination in groundwater.

Biodegradation was proved to be a cost-effective remediation technology for groundwater remediation in recent years. It was reported that a number of microorganisms have the ability to biodegrade EDB. *Dehalococcoides* and *Sulfurospirillum multivorans* could effectively biodegrade EDB under anaerobic conditions [12,13], whereas *Ancylobacter aquaticus* could biodegrade EDB under aerobic conditions [13]. Poelarends et al. [14] found that *Mycobacterium* sp. strain GP1 can utilize EDB as a sole carbon and energy source under aerobic conditions. Furthermore, some methanogenic strains were also reported to be able to improve the biodegradation efficiency of EDB [15]. 

Enhanced cometabolic degradation by adding co-substrates or nutrients to stimulate pollutant degradation activity has attracted broad attention due to the practicality of the field applications. It was found that cometabolism with phenol [16,17], ethane [16], and methane [2] under aerobic conditions, and lactate [2,18] under anaerobic conditions, could increase the biodegradation rate of EDB. However, it was observed that dissolved oxygen (DO) concentrations in groundwater often change dynamically between aerobic and anaerobic conditions during the engineering applications of enhanced reductive debromination of EDB in the field. In addition, DO concentrations were found, varying over a broad range from 0.1 to 8.7 mg/L, in natural groundwater [19,20,21]. Therefore, it is necessary to study the biodegradation of EDB under dynamic DO conditions to address the current research limitation focusing on either the aerobic or anaerobic biodegradation of EDB. 

In this study, in addition to the dynamic DO conditions, the influence of temperature, pH, and biomass of microbial consortium on EDB biodegradation were also studied. The addition of co-substrates (yeast extract, glucose, and sodium lactate) and a representative surfactant (rhamnolipid) were evaluated to further investigate possible approaches for enhancing EDB biodegradation efficiency. In addition, potential EDB biodegradation mechanisms by the consortium were explored. 

## 2. Materials and Methods 

### 2.1. Chemicals

Analytical grade EDB (99%) was purchased from Lingfeng Chemical Reagent Co., Ltd. (Shanghai, China). Sodium dihydrogen phosphate monohydrate (NaH_2_PO_4_-H_2_O, 99%), ammonium chloride (NH_4_Cl, 99.5%), magnesium sulfate heptahydrate (MgSO_4_-7H_2_O, 99%), zinc sulfate heptahydrate (ZnSO_4_-7H_2_O, 99.5%), and manganese (II) sulfate monohydrate (MnSO_4_-7H_2_O, 99%) were of analytical grade and were purchased from Xilong Scientific Co., Ltd. (Shanghai, China). Dipotassium hydrogen phosphate trihydrate (K_2_HPO_4_-3H_2_O, 98%), ferrous sulfate heptahydrate (FeSO_4_-7H_2_O, 99%), vitamin B_12_ (C_63_H_88_CoN_14_O_14_P, 99%), glucose (C_6_H_12_O_6_, AR), and yeast extract (BR) were purchased from Sinopharm Chemical Reagent Corporation Co., Ltd. (Beijing, China). Nitrilotriacetic acid trisodium salt monohydrate (N(CH_2_CO_2_Na)_3_-H_2_O, 98%) was obtained from TCI Development Co., Ltd. (Shanghai, China). Cobalt chloride hexahydrate (CoCl_2_-6H_2_O, 99%) was obtained from Tianjin Kemiou Chemical Reagent Co., Ltd. (Tianjin, China). Sodium lactate (C_3_H_5_NaO_3_, 60%) was obtained from Aladdin Chemistry Co., Ltd. (Shanghai, China). Rhamnolipid (>90% purity) was purchased from Sigma-Aldrich Co., Ltd. (St. Louis, MO, USA).

### 2.2. Cultivation of Microorganisms

The microbial consortium was acquired by acclimating anaerobic activated sludge, which was taken from the anaerobic tank of a sewage treatment plant in Nanjing. The activated sludge was acclimated through gradient culture in serum bottles with sterile mineral salts medium (MSM). The MSM contained the following constituents: 1.05 g/L K_2_HPO_4_-3H_2_O, 0.25 g/L NaH_2_PO_4_-H_2_O, 0.49 g/L NH_4_Cl, 0.03 g/L N(CH_2_CO_2_Na)_3_-H_2_O, 0.05 g/L MgSO_4_-7H_2_O, 0.003 g/L FeSO_4_-7H_2_O, 0.74 mg/L MnSO_4_-7H_2_O, 0.74 mg/L ZnSO_4_-7H_2_O, 0.25 mg/L CoCl_2_-6H_2_O, and 0.05 mg/L Vitamin B_12_ [7]. The initial pH of MSM was ~6.5–7.0. The MSM was purged with nitrogen gas by a nitrogen blowing concentrator to achieve a DO level of ~2.0 mg/L, and then sterilized in an autoclave at 121 °C for 30 min before use. 

The cultivation experiment was conducted in an anaerobic glove box (YQX-II) purchased from Xinmiao Medical Equipment Manufacturing Co., Ltd. (Shanghai, China), with the oxygen level set at ~2.0 mg/L. Two hundred milliliters of activated sludge and 700 mL of MSM were transferred into 1000 mL serum bottles. A 2 g/L EDB stock solution was prepared by dissolving a pre-calculated amount of EBD into deionized water. Each bottle was then dosed with the EDB stock solution, which was used as the sole carbon source in the cultivation. Serum bottles were sealed with Teflon-faced rubber septa and aluminum crimp caps made by Agilent (California, America) to minimize the EDB loss due to volatilization. The incubation was conducted at 25 °C in the dark in a thermostatic oscillator (MQL-621R) purchased from Minquan Instrument Co., Ltd. (Shanghai, China). The EDB concentration gradients of 1, 1, 2, 2, 3, 4, 5, and 10 mg/L were used as an enriched culture. EDB sampling was generally conducted on a weekly basis. Silicone caulking was used to seal the pinhole left on the cap when sampling to prevent EDB volatilization. 

After three months of culturing and acclimating, the composition of the enriched microbial consortium (EDB-degrading consortium) was identified by the 16S rRNA sequence analysis, and the EDB-degrading consortium was used in the microcosm study.

### 2.3. Microcosm Study

Batch experiments were conducted with an initial EDB concentration of 0.1, 0.5, and 1 mg/L in 120 mL serum bottles containing 100 mL of sterile medium and 20 mL of EDB-degrading consortium. Control experiments with EDB only in the medium were included. To simulate a relevant in situ groundwater condition, the incubation was generally conducted at pH values of 6.5–7.0, a DO of 2.0 mg/L, and a temperature of 15 °C in a thermostatic oscillator, unless specified otherwise. Samples were taken from the bottles periodically to determine the EDB concentrations. Resazurin, an indicator of oxygen content, was used to visually determine the dynamic changes of DO during the EDB biodegradation. The by-products of EDB, including bromide ion (Br) in aqueous solution, ethylene, methane, and carbon dioxide in gas phase, were taken at both the initial and the end of one set experiment for analysis. 

In the following batch experiment, an initial EDB concentration of 1 mg/L was used, to represent an actual concentration in the field [3,7]. The impacts of the environmental factors, including temperature, pH, DO, and biomass of the microbial consortium on the biodegradation efficiency, were investigated in this study. The range of temperature evaluated was 15–35 °C. All other experiments were conducted at 25 °C to expedite the evaluation process. The pH testing range was 6.0–8.0. The DO values were 1.2, 3.0, 5.1, and 7.8 mg/L. The biomass of microbial consortium was controlled by adding a predetermined MSM and assessed by the optical density at 600 nm (OD_600_) values of 0.12, 0.14, and 0.19. In the batch microcosm study, the killed control experiment was conducted using samples pre-autoclaved at 121 °C for 30 min.

The impacts of the co-substrates, including yeast extract, glucose, and sodium lactate, as additional carbon sources, and rhamnolipid as a representative of surfactant, were evaluated. Glucose and yeast extract were added at 20–160 mg/L, which was determined from a preliminary experiment. Sodium lactate was added at 0.1–0.8 mM [12], and rhamnolipid was evaluated at 20–100 mg/L [22]. Control experiments were conducted without the addition of co-substrates and surfactant. 

The efficiency of EDB biodegradation was calculated as follows:

(1) $$q=(C_{0}-C_{t})\times 100/C_{0}$$

where *q* (%) represents the biodegradation ratio, and *C*_0_ (mg/L) and *C_t_* (mg/L) are the initial and final concentrations of EDB, respectively. All of the experiments were performed in triplicate, and the mean values were used in the results discussion.

### 2.4. DNA Extraction and 16S rRNA Analysis

DNA extraction and purification were performed as described in other studies [23]. The region of 16S rRNA genes were amplified using the domain-specific universal forward primer 515F (5′-GTGCCAGCMGCCGCGGTAA-3′) and the universal reverse primer 806R (5′-GGACTACHVGGGTWTCTAAT-3′). Amplification was conducted by subjecting the samples to an initial denaturation step at 98 °C for 3 min, followed by 30 cycles of amplification (45 s denaturation at 98 °C, 45 s annealing at 55 °C, and 45 s extension at 72 °C) and then final extension at 72 °C for 7 min, after which the final solution was stored at 4 °C and sent to the Beijing Genomics Institute (BGI) for emulsion-based clonal (emPCR) amplification and sequencing in virtue of qualified library [24]. The PCR products were purified with AmpureXPbeads (AGENCOURT, USA Scientific, Ocala, FL, USA) to remove the unspecific products. Relevant biological information mainly related to species taxonomy, abundance and sample complexity were determined based on data acquired [25]. The final library was quantified by determining the average molecule length using an Agilent 2100 bioanalyzer instrument (Agilent DNA 1000 Reagents) and by quantifying the Library Purification Library Quality Control library with real-time quantitative PCR (qPCR) (Agilent, Santa Clara, CA, USA). The reads were processed using the SILVA reference database for bacterial sequences [26]. The qualified libraries were then pair-end sequenced on the MiSeq System, with the sequencing strategy PE250 (Illumina, San Diego, CA, USA). 

### 2.5. Analytical Methods

EDB concentrations were analyzed with gas chromatography (GC) spectrometry (Agilent 7820 A) equipped with an auto-sampler [2], a capillary column (DB-624, 30 m × 0.320 mm × 0.25 μm, Agilent, Santa Clara, CA, USA), and an electron capture detector. N_2_ was applied as a carrier gas at a constant flow rate of 1 mL/min. The injector and detector temperature were maintained at 250 and 300 °C, respectively. The oven temperature started at 50 °C, was held for 2 min, and was then increased to 140 °C at 15 °C/min, after which it was held for 1 min. Subsequently, the temperature was increased to 240 °C and then held for 1 min. 

By-product ethylene in gas phase was quantified by injecting 1 mL sample into a gas chromatograph (Agilent 6850) equipped with a flame ionization detector (FID). A PLOT capillary column (30 m × 0.53 mm × 30 μm, Agilent, Santa Clara, CA, USA) was used. The initial oven temperature of 50 °C was held for 8 min, while the temperature of the FID and injector were 220 and 120 °C, respectively. Potential by-products methane and carbon dioxide in gas phase were quantified using a GC (Agilent 7890, Santa Clara, CA, USA) equipped with an FID. A HP-PLOT U capillary column was used for methane analysis, while a Porapak Q column (Agilent, Santa Clara, CA, USA) was used for carbon dioxide analysis. The initial oven temperature was 55 °C, which was held for 5 min. The final temperature of the FID and the injector were both 250 °C. The detection limits of ethylene, methane, and carbon dioxide were 0.5 × 10^−6^ mol/mol, 0.15 × 10^−6^ mol/mol, and 5 × 10^−6^ mol/mol, respectively. 

Biomass concentrations in solutions were estimated using the initial OD_600_ by the ultraviolet and visible spectrophotometry TU1810 (Persee, Beijing, China). Bromide ion in aqueous solution was analyzed using ion chromatography (ICS1100, DIONEX, Sunnyvale, CA, USA). DO was determined with a HQ40d Portable and Benchtop Meter Configurator (HACH Company, Loveland, CO, USA).

## 3. Results and Discussion

### 3.1. Composition of the Acclimated Microbial Consortium

**Figure 1:**
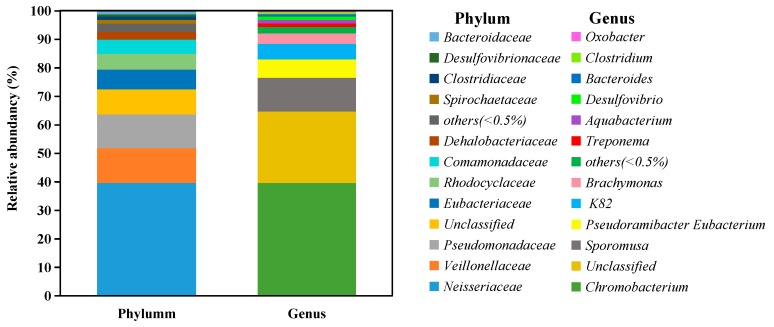
Composition of acclimated microbial consortium at the phylum and genus levels.

A total of 11 frequently detected bacterial phyla were identified in the acclimated microbial consortium, including *Neisseriaceae*, *Veillonellaceae*, *Pseudomonadaceae*, *Eubacteriaceae*, *Rhodocyclaceae*, *Comamonadaceae*, *Dehalobacteriaceae*, *Spirochaetaceae*, *Clostridiaceae*, *Desulfovibrionaceae*, and *Bacteroidaceae* (Figure 1). Among these, *Neisseriaceae* (accounting for 39.62%) predominated in the consortium, mainly consisting of *Chromobacterium*. This was followed by *Veillonellaceae*, which showed a proportion of 12.23% in the consortium, and then *Pseudomonadaceae*, which had an abundance of 11.82%. 

There were 11 frequently detected genera in the consortium (Figure 1). The previously reported EDB-degrading microorganisms, such as *Dehalococcoides* [12] or methanogenic bacteria [15], were not observed in this study. However, *Desulfovibrio* (1.17%), which could participate in dechlorination and sulfate reduction of several halogenated contaminants, including 1,2-dichloroethane [27], penta-brominated diphenyl ethers [28], 1,1,1-trichloroethane [29], and chloroform [30], was found in the consortium. In addition, *Clostridium*, which was shown capable of degrading a variety of volatile organic compounds, including tetrachloromethane, vinyl chloride, 1,2-dichloroethylene, trichloroethylene (TCE), tetrachloroethylene, 1,2-dichloroethane, 1,1,2-trichloroethane, and 1,1,1-trichloroethane [31], was found in the consortium, with a relative abundancy of 0.61%. This indicated that *Desulfovibrio* and *Clostridium*, even at relatively low abundance, may contribute to EDB biodegradation. 

Some other critical genera that might be responsible for EDB degradation were also observed in the consortium, mainly including *Chromobacterium* (39.57%), *Sporomusa* (11.84%), *Pseudoramibacter Eubacterium* (6.45%), K82 (5.39%), *Brachymonas* (3.69%), *Treponema* (1.30%), and *Aquabacterium* (1.25%). Previous studies showed that these genera could degrade organic pollutants. For example, *Chromobacterium* is a facultative anaerobe that can grow under anaerobic conditions but is also able to survive in aerobic conditions. *Chromobacterium* is known to be able to biodegrade numerous environmental organic pollutants, such as halogenated alkanes [32], polycyclic aromatic hydrocarbons (PAHs) [33], and acyl homoserine lactone [34]. Members of the genus *Sporomusa* were linked to the biodegradation of various organic chemicals, including methanol [35] and lignin-derived methoxylated monoaromatics (vanillate and syringate) [36]. K82 was reported to be responsible for the biodegradation of benzoate and p-hydroxybenzoate, mainly induced by catechol 1,2-dioxygenase and protocatechuate 4,5-dioxygenase, respectively [37]. *Brachymonas* was able to degrade PAHs [38], and *Treponema* was the dominant strain during the pentachlorophenol dechlorination with acetate [39]. Aquabacterium was found to degrade oil [40], benzene [41], and methyl *tert*-butyl ether (MTBE) [42], while *Bacteroides* could degrade MTBE [43]. These genera could play important roles in the biodegradation process of EDB. 

### 3.2. EDB Biodegradation 

**Figure 2:**
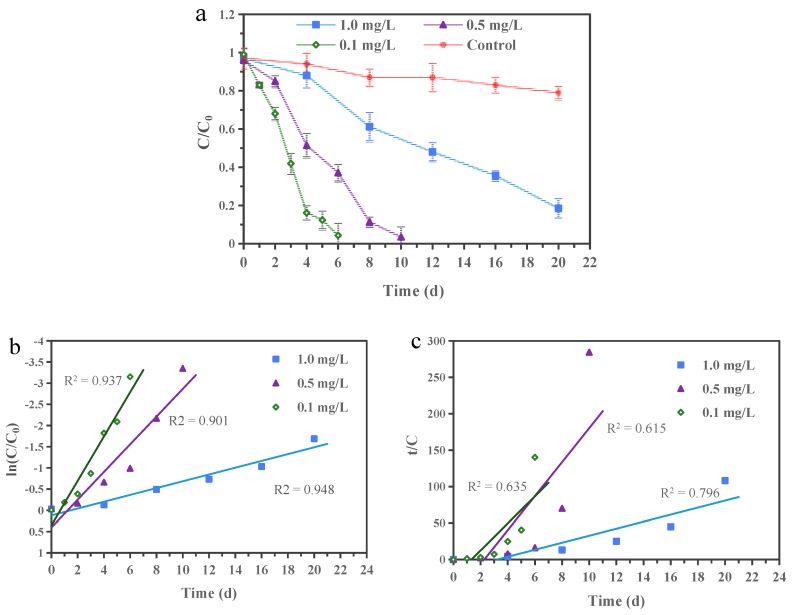
(**a**) EDB biodegradation kinetics and (**b**) its kinetic fittings by the pseudo-first-order, (**c**) pseudo-second-order in microcosms at 15 °C.

The EDB biodegradation kinetics are shown in Figure 2a. When compared to the EDB loss of 21% in the uninoculated control (primarily due to vaporization during the experiment), EDB was removed completely in 6 and 10 days when the initial concentrations were 0.1 and 0.5 mg/L, and 82% of the EDB was removed within 20 days at the initial concentration of 1.0 mg/L. The differences of more than 61% between the uninoculated control and those inoculated batch at different EDB concentrations were attributed to the biodegradation by the microbial consortium. In addition, it was observed that the color of the medium gradually changed from blue to pink and finally colorless, indicating that the EDB was biodegraded under a dynamic DO condition (from aerobic to anaerobic) by the acclimated consortium.

The EDB biodegradation efficiency under the relevant groundwater conditions in this study was compared with those observed in previous studies under aerobic or anaerobic conditions by a single species or mixed culture (Table 1). As shown, the majority of previous EDB biodegradation studies were conducted under aerobic conditions by indigenous microorganisms or mixed culture, with a few exceptions under anaerobic conditions [2,12,13]. The present study is the first to explore the potential of enhanced EDB biodegradation under a dynamic DO condition, which was often observed in the engineered field applications. More importantly, the relatively higher biodegradation efficiency in this study (>61% at 15 °C) indicated that the acclimated microbial consortium had a potential for in situ EDB bioremediation. 

Experimental data describing EDB biodegradation kinetics by the microbial consortium were fitted by applying two kinetic models: a pseudo-first-order model and a pseudo-second-order model (Figure 2b,c) [45,46]. The pseudo-first-order and the pseudo-second-order kinetic rate equations can be described mathematically as Equations (2) and (3) [47,48]:

(2) $$In(C/C_{0})=k_{1}t$$

(3) $$t/C=1/k_{2}C_{0}{}^{2}+t/C_{0}$$

where *C*_0_ (mg/L) and *C* (mg/L) are the EDB concentrations at the initial time and time *t* (d), *k*_1_ (d^−1^) is the pseudo-first-order rate constant for the biodegradation of EDB, and *k*_2_ (L mg^−1^ d^−1^) is the pseudo-second-order rate constant for the biodegradation of EDB. The experimental data fit the pseudo-first-order plot (r^2^ > 0.90) better than the pseudo-second-order plot (r^2^ = 0.62–0.80). Therefore, the EDB biodegradation can be described best by the apparent pseudo-first-order kinetics, and the biodegradation rate constants were 0.52, 0.33, and 0.08 d^−1^ at 0.1, 0.5, and 1.0 mg/L EDB, respectively. 

### 3.3. Influence of Environment Factors on EDB Biodegradation

**Figure 3:**
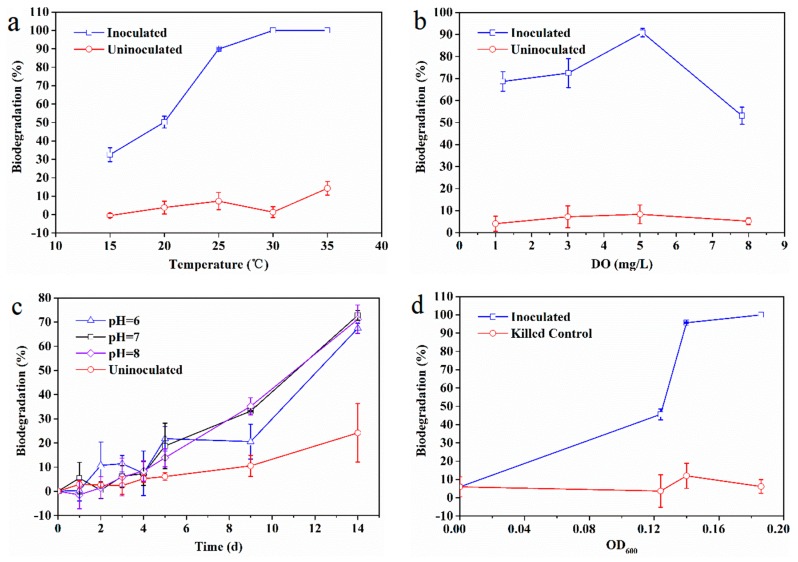
Effect of (**a**) temperature, (**b**) DO, (**c**) pH, and (**d**) biomass on EDB biodegradation by the microbial consortium at 25 °C.

The effects of temperature, DO, pH, and biomass on EDB biodegradation are presented in Figure 3. Temperature had an obvious influence on EDB biodegradation (Figure 3a). The EDB biodegradation rate increased from 33% at 15 °C to 90% at 25 °C. Complete biodegradation of EDB was observed at 30 and 35 °C, while the average EDB loss was generally within 7% in the uninoculated control. The main reason for the observed effect of temperature may be due to the enhanced activities of enzymes responsible for EDB biodegradation. Mukherjee and Roy [49] reported that the activity of enzymes responsible for TCE biodegradation was enhanced by the increase in temperature and that the maximum activity was seen at 36 °C.

The impact of DO on EDB biodegradation by the consortium was shown in Figure 3b. Some 70% EDB was degraded by the microbial consortium when the DO was 1.2 and 3.0 mg/L. The best biodegradation efficiency of 91% was observed in the presence of 5.1 mg/L DO, while it declined to 53% at 7.8 mg/L DO, indicating that the DO levels exert some influence on EDB biodegradation; however, a key finding is that the microbial consortium can adapt to different DO levels. It was also speculated that the influence of DO might be the sensitivity of some enzymes responsible for EDB biodegradation, resulting in the decrease of EDB biodegradation [50]. The broad adaptability of the microbial consortium differs from those of a previous study in which TCE reduction was significantly inhibited when DO was >0.5 mg/L [51]. This discrepancy might have occurred because the microbial consortium in the present study contained not only anaerobic bacteria (e.g., *Desulfovibrio* and *Eubacterium*), but also facultative anaerobes (e.g., *Chromobacterium*), which can adapt under a relatively wide range of DO. The results indicated that the microbial consortium investigated herein has the potential for in situ bioremediation applications.

The rate of EDB biodegradation over time remained the same at pH 6.0, 7.0, and 8.0, with about 20% EDB being degraded in the first five days (Figure 3c). During the next nine days, the biodegradation rate at pH 6.0 was slightly lower than at pH 7.0 and 8.0. These results indicated that a slightly acidic environment (pH < 7.0) and a slightly alkaline environment (pH > 7.0) did not have a significant impact on EDB biodegradation, suggesting that the microbial consortium had a good ability to degrade EDB in the pH range of 6.0–8.0. Similar optimal pH ranges for dechlorinating bacteria were observed in previous studies [31,52]. This can be attributed to the effect of pH on enzyme activity. Sfetsas et al. [53] found that the EDB-degrading haloalkane dehalogenase preserved 65% and 50% of its activity, in the pH ranges of 7.5–8.2 and 8.2–8.8, respectively; the enzyme activity dropped sharply to almost zero for pH values below and above these ranges.

The biodegradation efficiency increased with increasing OD_600_ (Figure 3d). The EDB biodegradation efficiency of 46% and 96% were observed at the corresponding initial OD_600_ values of 0.13 and 0.14, respectively, while it was completely degraded at an OD_600_ of 0.19. The EDB loss of a killed control set was significantly lower, confirming that the EDB loss in the microcosm study was mainly due to biodegradation rather than volatilization. 

### 3.4. Effects of Co-Substrates and Surfactant on EDB Biodegradation

**Figure 4:**
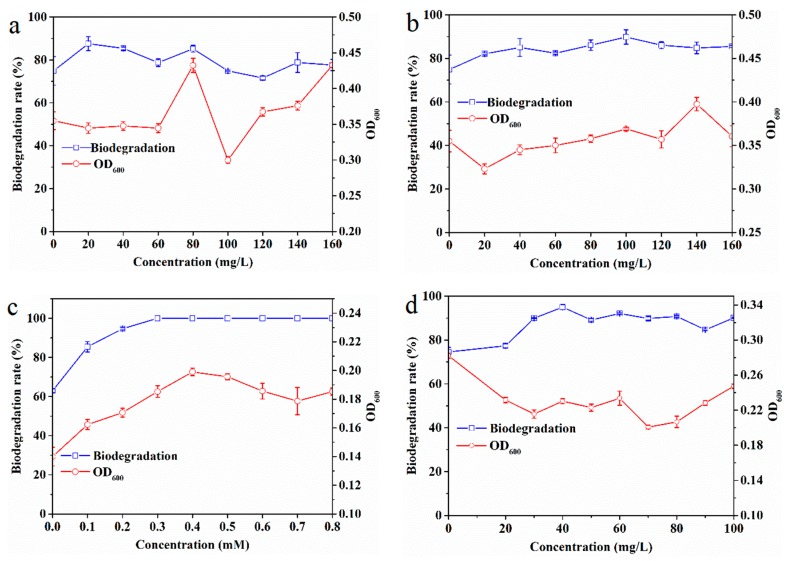
Effects of (**a**) glucose, (**b**) yeast extract, (**c**) sodium lactate, and (**d**) rhamnolipid on EDB biodegradation at 25 °C.

The effects of co-substrates and surfactant on biodegradation of EDB are shown in Figure 4. In addition, the biomass of the microbial consortium expressed as OD_600_ is also presented. The temperature of 25 °C was chosen to expedite the evaluation process because the biodegradation rate was much higher than that at 15 °C. 

The biodegradation rates of EDB under different glucose concentrations and their corresponding OD_600_ values are shown in Figure 4a. The results revealed that without the addition of glucose the biodegradation rate was 77%; however, the EDB biodegradation rate increased to 87% and 85% with the addition of glucose at 20 and 40 mg/L, respectively. Moreover, the OD_600_ did not change in response to the addition of 20–60 mg/L glucose, but it was significantly enhanced by the addition of 80–160 mg/L glucose, with the exception of 100 mg/L glucose. The enhanced degradation of EDB observed by the addition of glucose was not significant. One possible explanation is that the cells in the microbial consortium primarily metabolized the exogenous organic carbon when present at high levels, consequently decreasing the degradation of the toxic target contaminant, as observed in a previous study that glucose did not favor biodegradation of triphenyltin [54]. 

The EDB biodegradation rate under different yeast extract concentrations and the corresponding OD_600_ are shown in Figure 4b. The results showed that the EDB biodegradation rate increased from 76.7% without yeast extract to 90% at 100 mg/L yeast extract. However, the corresponding OD_600_ increased significantly within the range of 40–160 mg/L. This phenomenon may be because the yeast extract cannot induce the degrading enzymes, such as oxygenase enzymes, to promote EDB biodegradation [17], but can act as a carbon source for microbial growth. 

The biodegradation efficiency of EDB under different sodium lactate concentrations and the corresponding OD_600_ values are shown in Figure 4c. The results illustrated that the EDB biodegradation rate was 63% without the addition of sodium lactate, but the EDB biodegradation rate increased with increasing concentrations of sodium lactate. Complete biodegradation occurred at a concentration of 0.3 mM sodium lactate and the concentrations of sodium lactate above 0.3 mM. Therefore, 0.3 mM sodium lactate was sufficient for the microbial consortium to stimulate complete EDB biodegradation in this study. Given that the OD_600_ values corresponded to the EDB biodegradation efficiency under different sodium lactate concentrations, it can be concluded that sodium lactate expedites the possible role of the consortium in the EDB degradation by favoring the growth of the consortium. Similar results were also observed in a previous study in which sodium lactate was found to be one of the suitable electron donors for 1,2-dichloroethane [55] and PCE under anaerobic biodegradation [56].

We speculate that the potential mechanisms of co-substrates contribution under dynamic DO conditions are as follows. Under aerobic conditions, EDB may be catalyzed by oxygenase enzyme induced by different substrates, such as ethane and jet fuel, as used by Hatzinger et al. [7] and Baek et al. [57], respectively. Similarly, monooxygenases were induced by methane or ethanol to degrade chlorinated ethenes [58]. When the DO was consumed, anaerobic bacteria containing the genes of reductive dehalogenases such as *pceA*, *tceA*, *bvcA,* and *vcrA* began to function, and anaerobic dehalogenation occurred [59,60]. This interesting observation is key to understanding the phenomenon observed in the field application of EDB biostimulation.

The effects of rhamnolipid as a representative surfactant on EDB biodegradation are shown in Figure 4d. The results showed an overall positive impact of rhamnolipid on the EDB biodegradation. The addition of 20 mg/L rhamnolipid increased the EDB biodegradation efficiency to some extent but not significantly. However, the addition of rhamnolipid at concentrations ranging from 30 to 100 mg/L enhanced the EDB biodegradation from 76% in the absence of rhamnolipid to 85–95%, with the maximum EDB biodegradation observed at 40 mg/L rhamnolipid. This positive behavior in enhancing EDB degradation by adequate rhamnolipid addition is similar to the results of a study conducted by Bai et al. [22] in which it was suggested that rhamnolipid promoted biodegradation of carbendazim in a concentration-dependent manner, with the maximum biodegradation efficiency achieved in response to the addition of 50 mg/L rhamnolipid. As demonstrated by Lamichhane et al. [61], rhamnolipid can regulate hydrophobicity of the cell surface through which it will enhance the affinity of the degrading strain to EDB and consequently improve biosorption and metabolism, leading to a higher bioavailability of EDB. In addition to functioning as a biosurfactant, rhamnolipid could also serve as a nutrient for cell metabolism because of its bioavailability, which was partially responsible for the increased EDB biodegradation [62].

### 3.5. Potential EDB Biodegradation Pathways

**Figure 5:**
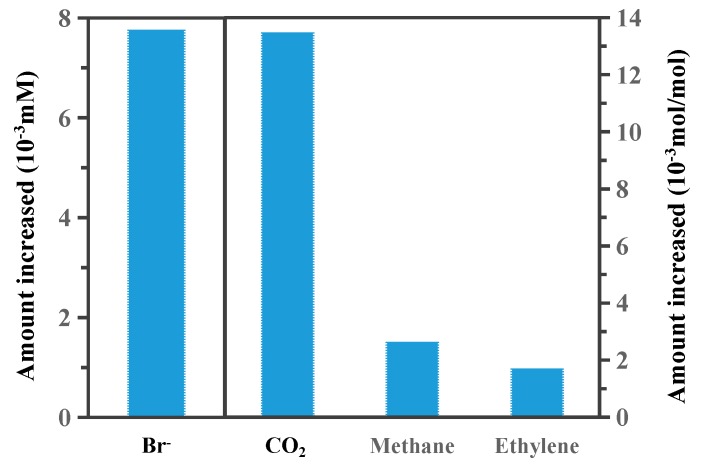
By-products of EDB biodegradation by the acclimated microbial consortium.

In this study, the composition of the aqueous and gas phases in the serum bottle before and after the reaction was analyzed to determine the mechanisms of EDB biodegradation by the acclimated microbial consortium (no co-metabolism matrix or biosurfactant, and EDB is the sole carbon source). As shown in Figure 5, Br concentrations increased in the aqueous solution, and the difference in concentration between before and post incubation was 7.75 × 10^−3^ mM. In addition, the changes in the concentration of ethylene, carbon dioxide, and methane were 1.68 × 10^−3^, 13.46 × 10^−3^, and 2.61 × 10^−3^ mol/mol, respectively, which were calculated to be statistically significant (*p* < 0.05). The detections of these by-products confirmed that EDB biodegradation occurred in this study. 

It was reported that, under aerobic conditions, EDB could be biodegraded to 2-bromoethanol as an intermediate product, which was rapidly converted to ethylene oxide via nucleophilic substitution (Equation (4)) [13,14]. However, the degradation pathway of ethylene oxide that resulted in the production of carbon dioxide was still unclear [13], as illustrated in Equation (4). The detection of carbon dioxide in the gas phase in this study suggested that EDB biodegradation occurred under aerobic conditions in the early phase of the experiment. The low DO in the medium was gradually consumed until it became completely anaerobic when methane production occurred. A previous study showed that EDB could not be biodegraded to methane directly [63]; however, as the production of methane was confirmed in this study, we speculated that the intermediate products (such as 2-bromoethanol or ethylene oxide) or other by-products may contribute to the production of methane under anaerobic conditions.

(4) $$C_{2}H_{4}{\mathrm{Br}}_{2}+H_{2}{O----C}_{2}H_{4}\mathrm{BrOH}\overset{}{\to }C_{2}H_{4}{O----\mathrm{mineralization}\;(\mathrm{CO}}_{2})$$

It was found that under anaerobic conditions, the predominant pathway of EDB was dihaloelimination directly to ethene [12]. In addition, Yu et al. [12] determined that the hydrogenolysis of EDB yields minor amounts of bromoethane, which may be further reduced to ethane; and the dehydrohalogenation of EDB yields minor amounts of vinyl bromide (VB), which may undergo hydrogenolysis to ethene. Based on the increase in Br in the aqueous phase and the detection of ethene in the gas phase, and the absence of VB, it was inferred that EDB was converted into Br and ethylene in a two-electron transfer pathway of dihaloelimination (Equation (5)). This observation is consistent with the conclusions from the study of Kuntze et al. [13], in which the dual carbon-bromine stable isotope was used to determine the biotransformation pathways of EDB, and it was found that EDB was degraded to ethylene directly in response to *Sulfurospirillum multivorans* under anaerobic conditions.

(5) $$C_{2}H_{4}{\mathrm{Br}}_{2}+2[H]\overset{\mathrm{dihaloelimination}}{\to }C_{2}H_{4}+2\mathrm{HBr}$$

It is proposed that there are two phases of biodegradation in this study: aerobic and anaerobic biodegradation. This could be evidenced by the fact that *Chromobacterium*, the most abundant genera in the consortium, is a facultative anaerobe. The consortium started with biodegradation under aerobic conditions and EDB was oxidized to carbon dioxide. Over time, DO was gradually consumed, resulting in an anaerobic condition in the medium. EDB was converted into ethylene and bromide via a double-electron transfer pathway. It was speculated that methane might be produced from the intermediate products of EDB (e.g., bromoethanol or ethylene oxide); however, it does not exclude the possibility that the microbial self-degradation may contribute to the production of methane and carbon dioxide, though to a much less extent, especially considering the large amounts that were detected in this study.

## 4. Conclusions

In this study, an effective EDB-debromiding consortium consisting of anaerobic bacteria (e.g., *Desulfovibrio* and *Eubacterium*) and facultative anaerobes (e.g., *Chromobacterium*) was enriched. The results showed that the initial DO at 1.2–5.1 mg/L could promote the EDB biodegradation, while 7.8 mg/L of DO inhibited the biodegradation of EDB. Additionally, when the co-substrate of sodium lactate and rhamnolipid were at 0.3 mM and 40 mg/L, the EDB biodegradation efficiency was enhanced by 19% and 37%, respectively. Moreover, the detections of by-products, including Br, ethylene, methane, and carbon dioxide, confirmed that EDB biodegradation occurred both aerobically and anaerobically. Taken together, these results confirmed that the acclimated microbial consortium had the potential for use in in situ biodegradation of EDB. 

## Figures and Tables

**Table 1:** Comparison of observations of EDB biodegradation from the present work and other reports in the literature.

Organism	Culture	Temperature (°C)	Co-Substrate	Initial EDB Conc. (mg/L)	Time for Corresponding Degraded Rate (Days)	Degraded Rate	Reference
*Ancylobacter aquaticus AD20*	aerobic	28	No	187	-	-	[13]
*Sulfurospirillum multivorans*	anaerobic	28	No	187	-	-	[13]
*Dehalococcoides* sp.	anaerobic	23 ± 1	lactate	2.2	20	under MCL ^2^	[12]
Mixed culture	aerobic	-	benzene	6.6	4	completely	[44]
Mixed culture	aerobic	-	pentane	6.6	8	0	[44]
Mixed culture	aerobic	-	toluene	6.6	8	0	[44]
Indigenous microorganisms	aerobic	14 ± 1	ethane	0.6	0.16	75%	[7]
Indigenous microorganisms	aerobic	24 ± 1	ethane	0.06	65	completely	[7]
Indigenous microorganisms	aerobic	14 ± 1	methane + DAP ^1^	0.06	56	60%	[7]
Indigenous microorganisms	aerobic	24 ± 1	phenol	0.06	56	20%	[7]
Indigenous microorganisms	aerobic	24 ± 1	propane	0.06	65	completely	[7]
Indigenous microorganisms	aerobic	12 ± 2	phenol	0.01	200	80%	[17]
Indigenous microorganisms	aerobic	12 ± 2	methane	0.08	≥230	completely	[2]
Indigenous microorganisms	anaerobic	12 ± 2	No	0.05	282	85%	[2]
Indigenous microorganisms	anaerobic	12 ± 2	lactate	0.09	282	88%	[2]
Microbial consortium	From aerobic to anaerobic (initial DO: ~2 mg/L)	15	no	0.1–1.0	6–20	>61%	present study

^1^ DAP: diammonium phosphate. ^2^ MCL: maximum contaminant level.
